# Red Blood Cells Protein Profile Is Modified in Breast Cancer Patients

**DOI:** 10.1016/j.mcpro.2022.100435

**Published:** 2022-10-28

**Authors:** Thais Pereira-Veiga, Susana Bravo, Antonio Gómez-Tato, Celso Yáñez-Gómez, Carmen Abuín, Vanesa Varela, Juan Cueva, Patricia Palacios, Ana B. Dávila-Ibáñez, Roberto Piñeiro, Ana Vilar, María del Pilar Chantada-Vázquez, Rafael López-López, Clotilde Costa

**Affiliations:** 1 Roche-Chus Joint Unit, Translational Medical Oncology Group, Oncomet, Health Research Institute of Santiago de Compostela (IDIS), Santiago de Compostela, Spain; 2 Proteomic Unit, Instituto de Investigaciones Sanitarias-IDIS, Complejo Hospitalario Universitario de Santiago de Compostela (CHUS), Santiago de Compostela, Spain; 3 CITMAga, University of Santiago de Compostela (Campus Vida), Santiago de Compostela, Spain; 4 Department of Oncology, University Hospital of Santiago de Compostela (SERGAS), Santiago de Compostela, Spain; 5 CIBERONC, Centro de Investigación Biomédica en Red Cáncer, Madrid, Spain; 6 Department of Gynecology, University Hospital of Santiago de Compostela (SERGAS), Santiago de Compostela, Spain

**Keywords:** red blood cells, breast cancer, metastasis, biomarkers, LAMP2, RDW, BC, breast cancer, CFC, cancer-free controls, CTCs, circulating tumor cells, GSEA, gene set enrichment analysis, Hb, hemoglobin, M0, nonmetastatic, M1, metastatic, MS, mass spectrometry, PBMCs, peripheral blood mononuclear cells, PNP, purine nucleoside phosphorylase, RBCs, red blood cells, RDW, red blood cell distribution width, SWATH-MS, Sequential Window Acquisition of All Theoretical Mass Spectra

## Abstract

Metastasis is the primary cause of death for most breast cancer (BC) patients who succumb to the disease. During the hematogenous dissemination, circulating tumor cells interact with different blood components. Thus, there are microenvironmental and systemic processes contributing to cancer regulation. We have recently published that red blood cells (RBCs) that accompany circulating tumor cells have prognostic value in metastatic BC patients. RBC alterations are related to several diseases. Although the principal known role is gas transport, it has been recently assigned additional functions as regulatory cells on circulation. Hence, to explore their potential contribution to tumor progression, we characterized the proteomic composition of RBCs from 53 BC patients from stages I to III and IV, compared with 33 cancer-free controls. In this work, we observed that RBCs from BC patients showed a different proteomic profile compared to cancer-free controls and between different tumor stages. The differential proteins were mainly related to extracellular components, proteasome, and metabolism. Embryonic hemoglobins, not expected in adults’ RBCs, were detected in BC patients. Besides, lysosome-associated membrane glycoprotein 2 emerge as a new RBCs marker with diagnostic and prognostic potential for metastatic BC patients. Seemingly, RBCs are acquiring modifications in their proteomic composition that probably represents the systemic cancer disease, conditioned by the tumor microenvironment.

## Highlights

• The red blood cell (RBC) proteins are modified due to the presence of a breast tumor.
• Embryonic or fetal hemoglobins were found in RBCs from breast cancer patients.
• LAMP2 from RBCs is a prognostic biomarker in breast cancer patients.
• RBCs display unexplored functions in the metastatic setting.

## Graphical Abstract

**Figure:**
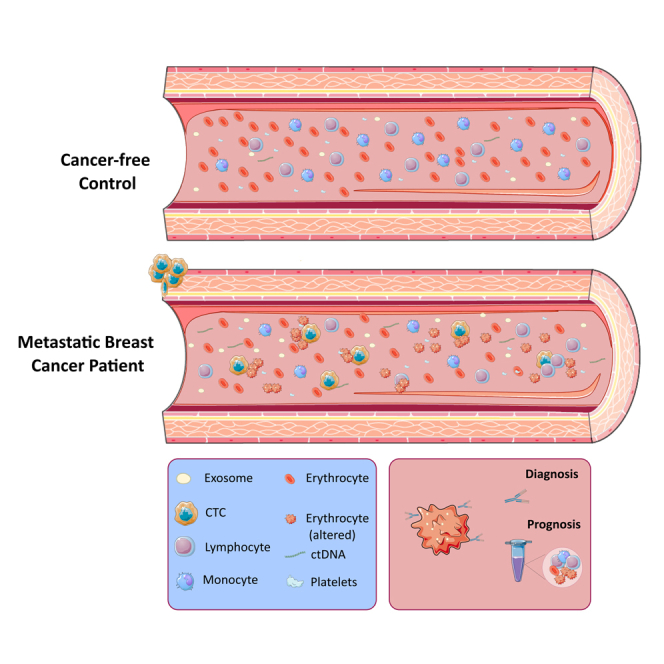

## In Brief

This study demonstrates for the first time that the protein composition of red blood cells is different in the presence of a breast tumor, providing new information on the potential role of RBCs in the context of disseminated disease. In addition, it points to the value of red blood cell proteins in the prognosis and diagnosis of metastatic breast cancer in a non-invasive way.

Breast carcinoma is the leading cancer-related cause of death in women and has a higher incidence rate than any other type of tumor (https://gco.iarc.fr/). The metastatic disease accounts for the overwhelming majority of cancer-related deaths; thus, the lack of reliable biomarkers for early metastasis diagnoses remains as one of the main clinical challenges. Tumor metastasis involves a multistep process where cancer cells escape from their primary site, circulate in the bloodstream as circulating tumor cells (CTCs), and then extravasate through the vascular walls into the parenchyma of distant tissues, where they finally adapt and outgrowth (1, 2). Once CTCs reach and settle in a distant organ, they are called disseminated tumor cells and together with CTCs are recognized as the seeds of metastasis (3, 4). Interactions between CTCs and normal blood components such as platelets, neutrophils, monocytes, and endothelial cells are crucial for their survival in the bloodstream and can facilitate the extravasation at distant sites (5). Surprisingly, little is known about the role of the most abundant component of the blood, the red blood cells (RBCs), in the metastatic process.

RBCs account for ∼84% of the total blood cells count in the average adult (6). Erythropoiesis begins with the differentiation of multipotent hematopoietic stem cells in the bone marrow, which then give rise to erythroid-committed precursors. In the last stages of the process, the nucleus and other organelles are extruded, and these enucleated reticulocytes are released into the bloodstream to complete their maturation progress in a tightly regulated process. Related to erythropoiesis, abnormal red blood cell distribution width (RDW) has been associated with poor prognosis in cancer (7, 8, 9) and advanced disease (10, 11, 12, 13, 14, 15). Besides, our group has recently reported that the presence of escort RBCs in the enriched CTCs fraction was linked with a worse outcome on metastatic breast cancer (BC) patients (16). This observation suggested alterations in the RBCs of patients with metastatic BC.

This study aimed to provide for the first time a large-scale proteomic analysis of RBCs from cancer-free controls (CFCs) and BC patients. We extensively analyzed CFC and treatment-naïve metastatic and nonmetastatic BC patients using two proteomic approaches (shotgun and Sequential Window Acquisition of All Theoretical Mass Spectra [SWATH-MS]) to identify potential differences in proteomic profiles in BC patients’.

## Experimental Procedures

### Patients and Samples

Blood samples and associated clinical information were obtained at the University Hospital Complex of Santiago de Compostela (Spain). All patients and cancer-free controls (CFCs) gave written informed consent. All samples were anonymized. The study was conducted according to the guidelines of the Declaration of Helsinki and approved by the Ethics Committee of Galicia with approval reference number 2015/772.

In total, 126 blood specimens from 80 BC patients and 46 CFC were collected for this study with an average age of 57 years (29–83 years). The median age among patients and controls was similar (*t* test, *p* > 0.05). The CFC had different illness including diabetes (n = 4), hypertension (n = 5), fibromyalgia (n = 4), and/or osteoarthritis (n = 3). Patients’ clinical information is summarized in Table 1.

**Table 1:** Clinicopathologic characteristics of the BC patients and cancer-free controls

Category	M0		M1		CFC	
	Media	SD	Media	SD	Media	SD
Age	55.98	12.32	57.77	11.21	57	11.25
	n	%	n	%	n	%
Tumor stage						
0					46	36.51
I–II	17	13.49				
I–III	27	21.43				
IV			36	28.57		
Subtype						
Luminal	33	75	28	77.77		
Her2	2	4.54	1	2.77		
Triple negative	9	20.45	7	19.4		
Metastasis location						
Bone			25	69.44		
Visceral			25	69.44		
Bone & visceral			16	44.44		
Number of metastatic sites						
1			8	22.22		
2			10	27.77		
≥3			18	50.00		

### Samples Preparation

Ten ml of blood (EDTA tube) were centrifuged (1700*g*/10′), plasma and peripheral blood mononuclear cells (PBMCs) were discarded. From the RBCs fraction, 1 ml was lysed in 40 mM Hepes (Sigma-Aldrich), 2% Triton-x100 (Sigma-Aldrich), 200 Mm NaCl, 40 mM MgCl_2_, and 20 mM EGTA (Sigma-Aldrich), and 80 mM β-glycerophosphate (Sigma-Aldrich) and centrifuged to obtain supernatant containing total protein. Total protein quantitation was performed with DC Protein Assay following the manufacturer's instructions (Bio-Rad). Protein concentration and hematocrit obtained for each sample is listed on supplemental Table S1.

To test the presence of other blood cell populations unspecifically isolated on the RBCs fraction, randomly assays of microscopy and FACS were performed as quality control. The mean percentage of PBMCs and platelets on the RBC fraction were 0.014 and 0.053, respectively.

One additional EDTA tube was used for blood test analysis and processed in the clinical laboratory of the Oncology Department by standard assays.

### Proteomic Analysis by TripleTOF 6600 LC-MS/MS System

#### Protein Digestion

To make global protein identification, an equal amount of protein from RBCs of 86 samples (BC patients n = 53, CFC: n = 33) was loaded on a 10% SDS-PAGE gel to concentrate the proteins in a band. The band was processed as described on Supplementary Methods and Materials and previously (17, 18).

#### LC-MS/MS in Data-Dependent Acquisition Mode-Shotgun Analysis

Digested peptides of all individual samples from RBCs were separated using reverse phase chromatography as described previously (17, 18).

#### Protein Quantification by SWATH-MS Analysis

To build the MS/MS spectral libraries, the peptide solutions were analyzed by a shotgun data-dependent acquisition (DDA) approach using micro-LC-MS/MS as described (17). The MS2 spectra (MS/MS spectra) of the identified peptides were then used to generate the spectral library for SWATH peak extraction using the add-in for PeakView Software (version 2.2, Sciex), MS/MS^ALL^ with SWATH Acquisition MicroApp (version 2.0, Sciex). Peptides with a confidence score above 99% (as obtained from Protein Pilot database search) were included in the spectral library. For relative quantification by SWATH-MS analysis, SWATH-MS acquisition was performed on a TripleTOF 6600 LC-MS/MS system (Sciex). Peptides from RBCs samples from all CFC and BC patients were analyzed using the data-independent acquisition method making three technical replicates per sample as previously described (17, 18) and can seen in Supplementary Methods and Materials.

### Flow Cytometry

One EDTA tube of 7.5 ml was centrifuged at 1700*g* for 15 min. After centrifugation, plasma and PBMCs were removed. Fifteen microliter of concentrated RBCs were transferred and suspended in 3 ml of PBS into a cytometer tube. Samples were analyzed using a FACSAria Ilu cytometer (BD Biosciences) using 169 V for forward scatter and 300 V for side scatter. The sorting was performed considering changes in width in the RBC populations of BC patients with respect to a CFC sample.

### ELISA Assay

Total RBC protein, extracted as previously mentioned, was used for the determination of lysosome-associated membrane glycoprotein 2 (LAMP2). The ELISA assay (ABclonal) was performed following the manufacturer's recommendations using a 1:100 dilution. LAMP2 concentration values obtained were normalized with total protein concentration values.

### Statistical Analysis

Statistical analysis was performed using GraphPad Prism 6.01 software (GraphPad Software Inc) and R Studio (Version R-3.6.3). Wilcoxon signed-rank test was used for media comparisons and Fisher exact test or Chi-square test for association analysis. Progression-free survival and overall survival were visualized using Kaplan–Meier plots and tested by the log-rank test. Only *p*-values <0.05 were considered statistically significant.

The normalization and differential expression analysis of the proteomic data were performed using the Bioconductor NormalyzerDE package (19). The variance stabilization normalization method (20) and type limma (21) differential expression analysis was chosen among the different normalization options provided by the package. Proteins with Benjamini–Hochberg correction adjusted *p*-value <0.01 were considered as differential expressed.

To fit the logistic regression models, the glm function of the stats package of R was used. The subsequent analysis of the ROC curve was performed using the pROC package of R (22).

For gene set enrichment analysis (GSEA), Gene Ontology (GO) analysis, and protein interaction, the used tool was GO-Shiny V0.741 or String (https://string-db.org/), considering strength >1.2, >3 proteins and false discovery rate (FDR) <0.05. Venn diagrams were performed using http://www.interactivenn.net/ (23).

## Results

### Differential RBCs Proteomic Profiles Between BC Patients and Cancer-Free Controls

**Fig. 1:**
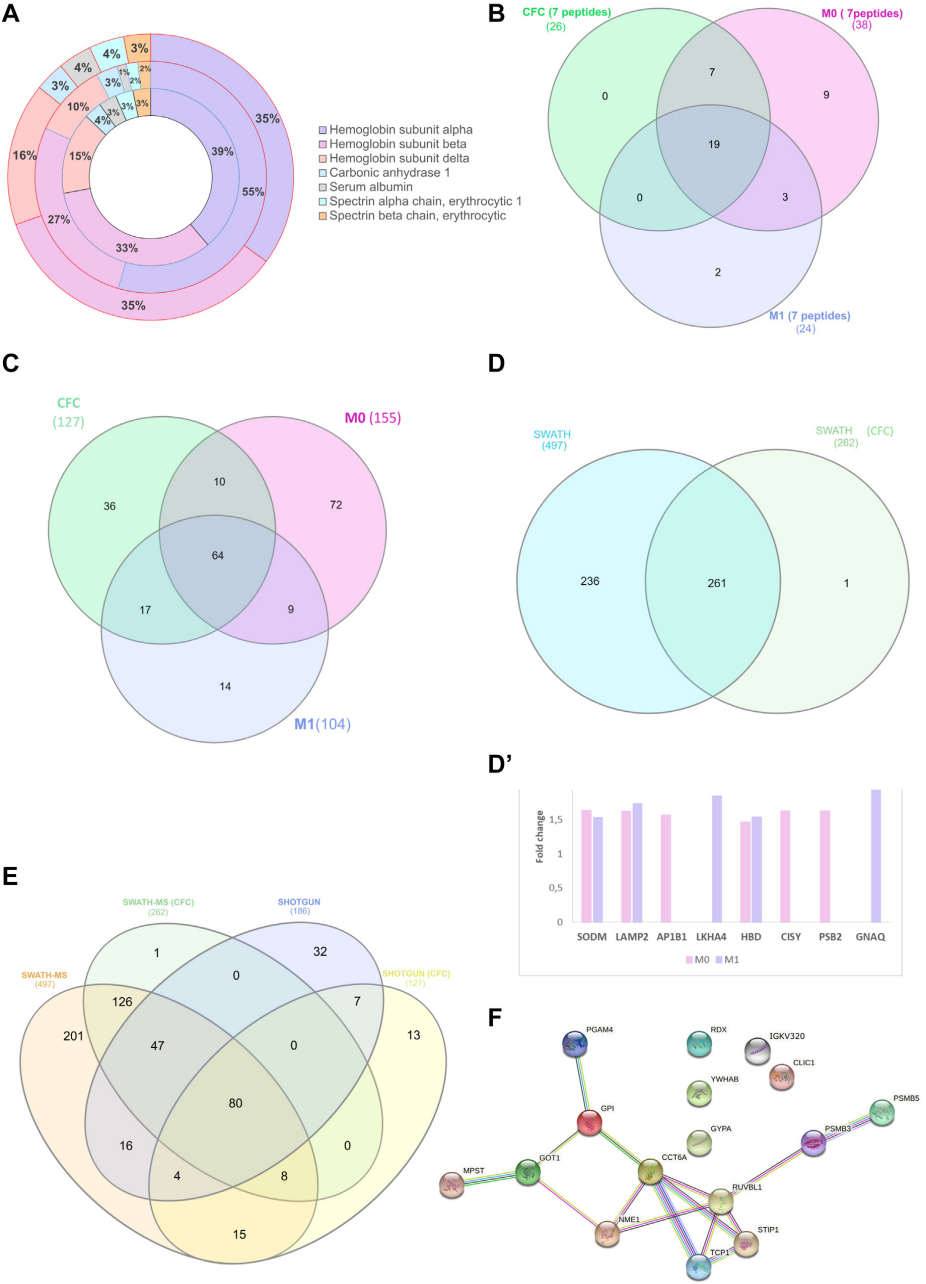
**Massive proteomic analysis of RBCs from BC patients and CFC.***A*, representation of the number of major peptides detected by shotgun in cancer-free controls (CFC) (the *inner black circle*), M0 patients (the *intermediate blue circle*), and M1 patients (the *external red circle*). *B* and *C*, Venn diagram showing the common proteins among patients (M0 and M1) and CFC RBCs samples identified using shotgun technology for ≥7 peptides (*B*) and ≥1 peptide (*C*). *D*, Venn diagram showing the common proteins among BC patients and CFC RBCs samples identified using SWATH-MS technology. *D'*, relative fold change of M0 and M1 compared to CFC of the indicated proteins. *E*, Venn diagram showing the common proteins among both approaches. *F*, string network analysis showing interactions between 16 common proteins identified by both approaches exclusively of BC patients. BC, breast cancer; M0, nonmetastatic; M1, metastatic; RBCs, red blood cells.

The proteomic analysis by LC-MS/MS in DDA mode-shotgun of RBCs of samples from BC patients (nonmetastatic: M0, n = 17; metastatic: M1, n = 19) and CFC (n = 21) (Table 1) mainly reported specific proteins of RBCs such as hemoglobins (Hbs) or spectrins (Fig. 1*A* and supplemental Table S2). However, the RBCs proteomic profile of BC patients resulted to be different from CFC.

We identified 14 proteins as unique to the patient samples when considering ≥7 peptides per protein and FDR <1% (Fig. 1*B* and supplemental Table S2, in gray). These proteins were associated with the metabolism of amino acids, glycolysis, and gluconeogenesis by KEGG pathways (PGK1, BPGM, ALDOA, and TPI1 proteins) (protein-protein interaction [PPI] enrichment *p*-value: 4.4e-07). In addition, the potentially related diseases to specific patients’ proteins were anemias or hematopoietic system diseases (proteins HBZ, EPB41, HP, PGK1, HBE1, BPGM, and ALDOA). Embryonic Hbs isoforms such as epsilon (HBE1) and zeta (HBZ) were identified exclusively in M1 and M0 patients, with 25 and 16 peptides, respectively.

Next, we checked all the detected proteins (≥1 peptide and FDR <1%) comparing CFC and BC patients (Fig. 1*C* and supplemental Tables S3–S5). M0 BC patients showed 72 differential proteins compared to CFC, among the 260 proteins that comprise the library. In M1 patients, 14 proteins were identified exclusively to the advanced stage. Nine proteins were shared between M0 and M1 BC patients. Considering the 95 proteins unique to the patients’ cohort, GO analysis pointed to proteasome and chaperone complex (KEGG pathways) (PPI enrichment *p*-value < 1.0e-16). Regarding the biological processes, regulation of amino acid metabolism or extracellular exosomes were highlighted. On these samples were also found proteins that are usually localized in Cajal bodies. No specific proteins of platelets, granulocytes, or lymphocytes were found on this analysis, proving the absence or negligible amount of other blood cells in the RBCs fraction of the analyzed samples. Besides, no erythroid precursor markers were observed.

Seeing that the RBCs of patients showed a different proteomic profile, we took advantage of the SWATH-MS strategy that enables quantitative analysis of proteins with high precision and consistency. The data output from CFC RBCs samples (n = 33) identified a total of 262 proteins (supplemental Table S6). The GSEA confirmed that the main enriched pathway was *erythrocytes take up carbon dioxide and release oxygen*. In agreement, one of the preferred tissues was RBCs.

Similar to shotgun data, proteomic profile from BC patients’ RBCs (M0, n = 26; M1, n = 27) was different to CFC (n = 33) by SWATH-MS (*t* test, *p* < 0.05) (Fig. 1*D*). The generated library including patients and CFC comprised 497 RBCs proteins (see Supplemental Information, PXD030936). Proteins found only in BC patients were related to integrin and inflammatory signaling pathways or extracellular matrix interaction and platelet degranulation. Next, we performed the quantitative analysis comparing CFC to both M0 and M1 BC patients. Compared to CFC, in M1 samples, 22 proteins were upregulated, 15 proteins were downregulated, while in M0 samples, ten proteins were upregulated, and 17 were downregulated (supplemental Table S7). Figure 1*D*' depicted those proteins that showed a greater fold change of expression in BC patients compared to controls, as GNAQ, LKHA4, or LAMP2. The GSEA of the proteins differentially expressed between patients and CFC emphasized the pentose phosphatase, biosynthesis of amino acids and, secretory granule lumen pathway (PPI enrichment *p*-value: <1.0e-16). In addition, proteins linked to congenital hemolytic anemia were also identified in these samples. Among the proteins differentially expressed in patients, no proteins specific to RBC precursors or other white blood cells were identified, except the platelets markers CD41 and CD61. BC patient's samples also showed differential protein expression based on the stage (M0 or M1). Thus, ten proteins were upregulated in M1 compared to M0, while seven proteins were downregulated (supplemental Table S7). Some of those proteins were also altered *versus* CFC (supplemental Table S7). These proteins were related with congenital hemolytic anemia (PPI enrichment *p*-value: 0.000359).

There was no association between the different subtypes of BC and the proteomic profile of the samples, although more than 75% of cases belonged to the luminal subtype, showing a significant imbalance among the subtypes.

#### Comparative Analysis Between Shotgun- and SWATH-MS Proteomics Data–Identified Common Proteins

The proteins identified by *shotgun* and SWATH-MS in these samples showed a high overlap, being 79% of identified proteins by *shotgun* also in SWATH-MS data (Fig. 1*E*). The GO analysis indicated similar pathways or biological processes, reinforcing the similarity in both approaches’ data. Of the proteins exclusive of BC patients, 16 matched in both approaches. These proteins are related to the chaperone complex and carbon metabolism (PPI enrichment *p*-value: 2.36e-06) (Fig. 1*F*).

### Identification of Nonreported Proteins in RBCs

**Fig. 2:**
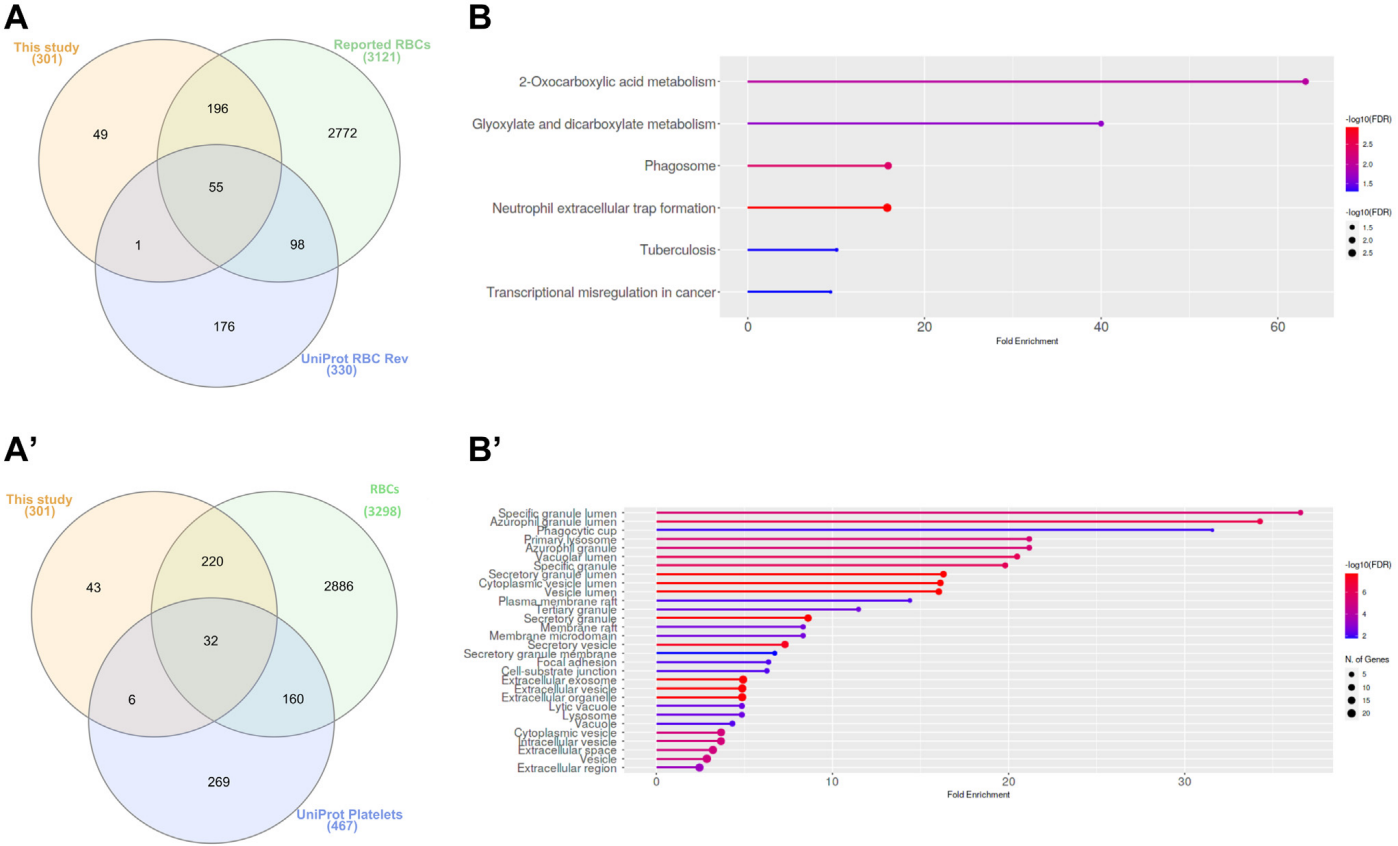
**Identification of potential novel RBCs proteins in cancer-free controls samples.***A* and *A'*, Venn diagram showing the common proteins between this study (shotgun or SWATH-MS), the reported proteins including the RESPIRE project (https://www.dsimb.inserm.fr/respire/), and two related publications (Bryk *et al.* and D'Alessandro *et al.*) (24, 25) and the UniProt RBCs database (revised) (*A*); or this study, RBCs (both reported and database), and UniProt platelets database (revised) (*A'*). *B* and *B'*, GO analysis of the 43 potential novel proteins identified regarding the KEGG pathway (*B*) and cellular components (*B'*) (ShinyGO v0.741). GO, Gene Ontology; RBCs, red blood cells.

The lists of identified proteins from CFC in this study by shotgun and/or SWATH-MS approaches were compared with the UniProt database for RBCs and the database Repository of Enhanced Structures of Proteins Involved in the Red Blood Cell Environment (RESPIRE). This latter database aims to provide a comprehensive reference of protein-based information on the proteins available in the RBCs. In addition, our data were compared with repositories from other RBCs publications (24, 25) and with the UniProt database for platelets (to verify that these proteins do not come from escorting platelets present on the RBC isolated sample). In total, 43 new proteins not previously described in RBCs or platelets databases were found in this study (Fig. 2, *A* and *A'* and supplemental Table S8). By GSEA, these proteins were mainly linked to primary lysosome, phagosome, and specific granule lumen, although specific markers associated with neutrophil (CD54, CD217, CD49d or CD11b) were absent (PPI enrichment *p*-value: <1.0e-16) (Fig. 2, *B* and *B'*).

### Metastatic BC Patients Showed Altered Blood Clinical Values

**Fig. 3:**
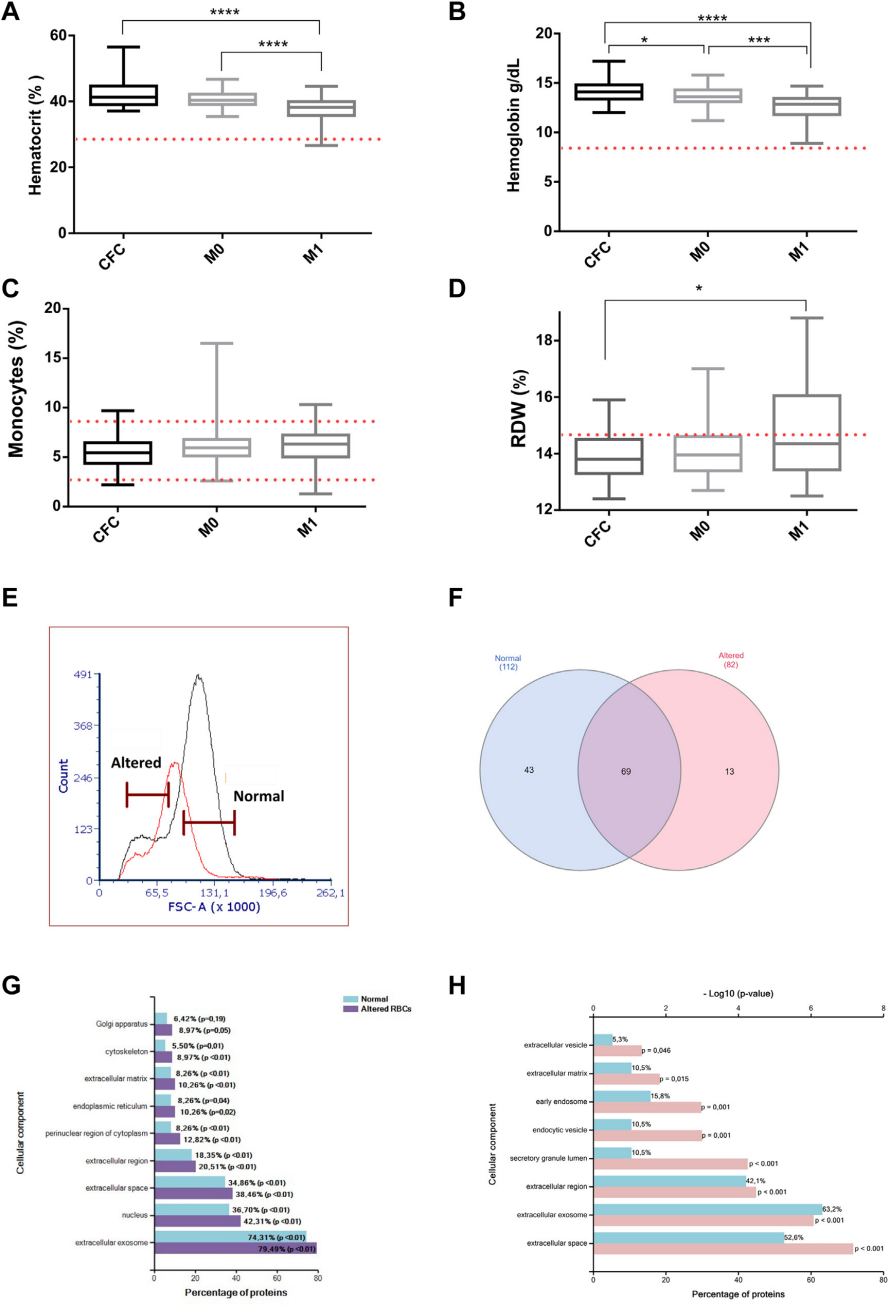
**Altered blood test parameters in nonmetastatic (M0) (n = 44) and metastatic (M1) (n = 34) breast cancer (BC) patients compared with cancer-free controls (CFC) (n = 30).***A*, the hematocrit represents the percentage of RBCs. The lower limit is 36.9%. M1 BC patients had a lower hematocrit compared to CFC or M0 patients. *B*, concentration of Hb in the blood. The lower limit is 12.2 g/dl. M1 BC patients had a lower concentration compared to CFC or M0 patients. Besides, M0 patients showed a lower level compared to CFC. *C*, the standard percentage range of monocytes is 2.7 to 8.6. M1 BC patients have a slight increase in monocytes compared with CFC, close to statistical significance. Only six patients had altered parameters from standard ones. *D*, red distribution width (RDW) represents the percentage of Red blood cells with abnormal size. The standard range is 11.5 to 14.5. M1 BC patients presented a high value compared with CFC. The *red dotted line* represents the limit of the standard value for each parameter. *p*-value < 0.05 (∗); *p*-value < 0.01 (∗∗); *p*-value < 0.001 (∗∗∗), and *p*-value < 0.0001 (∗∗∗∗). *E*, representative flow cytometry histogram depicting the forward scatter (FSC) of a CFC (*black line*) and M1 sample (*red line*). The CFC sample defines the normal and altered RBC population for the sorting assay. *F*, Venn diagram showing the common proteins between normal and altered sorted RBCs populations from two mBC patients by shotgun analysis. *G*, GO analysis showing the percentage of proteins for normal (in *blue*) and altered (in *purple*) RBC populations by shotgun. *H*, GO analysis of SWATH-MS data for those proteins overexpressed on the altered fraction compared to the normal fraction depicting the percentage of proteins (in *blue*) and the −log10 (*p*-value) that represents the level of significance of each gene (in *pink*). GO, Gene Ontology.

Blood cells indexes are included in all standard clinical tests. To check whether blood clinical parameters can be affected by the presence of BC or the tumor stage, these values were compared between the CFC and patients included in this study. Hematocrit and Hb concentrations were significantly lower in the M1 cohort (*p* < 0.0001, Kruskal–Wallis test) (Fig. 3, *A* and *B*). A slight increase of monocytes counts was observed on the M1 patients compared with CFC (*p* = 0.07, Mann–Whitney test) (Fig. 3*C*), while no differences were found for the count of lymphocytes, eosinophils, basophils, neutrophils, or platelets between the three groups. Interestingly, the levels of blood parameters of M0 patients were closer to CFC than to M1 BC patients.

The RDW parameter, which measures the variation in the volume and size of RBCs, was altered in the advanced disease. Thus, M1 patients showed higher values (mean 14.96 ± 1.92) compared with the M0 (mean 14.07 ± 0.93) or the CFC group (mean 13.88 ± 0.77) (Fig. 3*D*), (*p* = 0.01, Mann–Whitney test). Contingency analysis considering elevated (>14.5) or normal RDW (≤14.5) indicated that altered RDW was associated with M1 patients (*p* = 0.002, Fisher-exact test). No association was observed between the presence of bone metastasis and altered blood test levels, although the percentage of patients showing bone affectation is over 70%.

Next, since RDW alteration indicates changes on RBCs size, a proteomic comparative between different RBCs populations representing normal and altered RBCs from two different metastatic patients was performed. The selection of both populations was done by sorting considering a CFC sample as a standard (Fig. 3*E*). After sorting, both populations were analyzed by shotgun and SWATH-MS in the same way as previously described. As depicted in the Venn diagram (Fig. 3*F*), it was observed a differential protein profile with 43 and 13 proteins exclusively from the normal and altered fraction, respectively. In this proof of concept experiment, GO analysis showed that the altered RBCs population has an increase on the percentage of proteins on pathways mainly related with extracellular components or extracellular vesicles trafficking (Fig. 3, *G* and *H*) as previously seen between patients and CFC.

### LAMP2 as a Prognostic and Diagnostic Marker for Metastatic BC

**Fig. 4:**
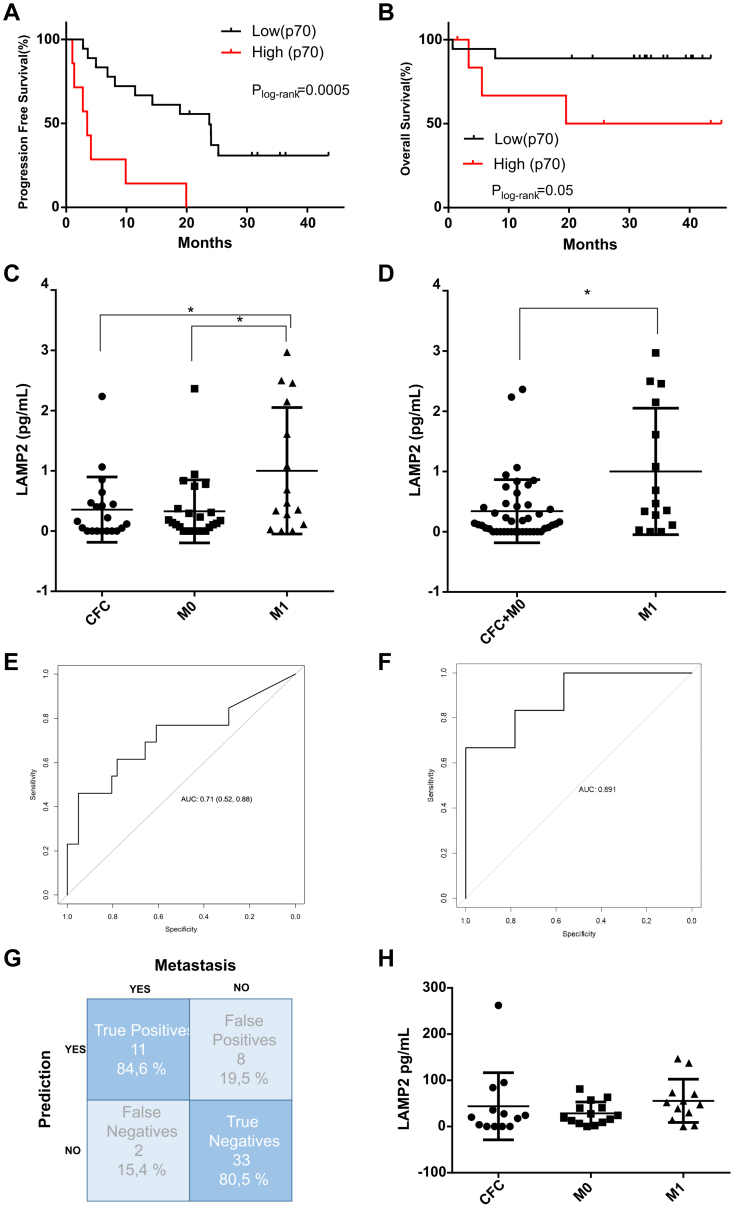
**LAMP2 as a prognostic and diagnostic marker for metastatic BC.***A* and *B*, Kaplan–Meier plot for PFS (*A*) and OS (*B*) of LAMP2 values (SWATH-MS data) for mBC patients (n = 27). Percentile 70 defines high or low levels of LAMP2. *C* and *D*, LAMP2 concentration levels from RBCs by ELISA assay in cancer-free controls (CFC, n = 20), nonmetastatic (M0, n= 24), and metastatic (M1, n = 15) (*C*), or nonmetastatic (M0 and CFC, n = 44) and metastatic (M1, n = 15) (*D*). Nonmetastatic patients were recruited presurgery. CFC samples were obtained from women with paired age to the patients. *p*-value < 0.05 (∗). *E*, ROC curve for LAMP2 concentration levels; AUC= 0.71. *F*, ROC curve for the predictive model using LAMP2 + hematocrit + RDW; AUC= 0.89. *G*, confusion matrix depicting the percentages of true positives and false negatives cases given by the model. *H*, LAMP2 concentration levels from plasma by ELISA assay in CFC, M0, and M1 (n = 13, each group). OS, overall survival; LAMP2, lysosome-associated membrane glycoprotein 2; PFS, progression-free survival; RBCs, red blood cells.

To check if the protein levels determined by SWATH-MS were linked with the patient's outcome, those proteins with a fold change higher than 1.5 in M1 patients *versus* CFC samples were selected (supplemental Table S7). To decipher the predictive potential of GNAQ, HBD, LKHA4, and LAMP2, a survival analysis was performed. The high or low protein expression levels were determined using the percentile 70. Protein levels of GNAQ, HBD, or LKHA4 did not show prognostic value in this cohort of study (log-rank test *p* > 0.05, n = 27). However, high LAMP2 expression levels can predict the worst outcome in M1 BC patients (progression-free survival: *p* = 0.0005, 3.5 *versus* 23.73 months, log-rank test; overall survival: *p* = 0.05, log-rank test) (Fig. 4, *A* and *B*). Besides, low levels of LAMP2 were found in patients without disease progression (*p* = 0.02, Chi-square test) or who were still alive during follow-up (*p* = 0.03, Fisher's exact test). Low LAMP2 levels were also associated with having normal RDW value (*p* = 0.02, n = 65: 18 CT, 22 M0, and 25 M1 samples). Altogether, the enhancement in LAMP2 levels was related to advanced disease. Interestingly, the purine nucleoside phosphorylase (PNP), whose specific enzymatic activity is very high in late erythroblast and RBCs, showed lower levels in patients with bone metastases (*p* = 0.02, Fisher's exact test).

LAMP2 is a lysosome-related membrane glycoprotein that has been associated with BC tumor cells. However, its function in RBCs is unknown. The higher LAMP2 levels observed on RBCs could indicate a systemic alteration due advanced disease and, therefore, may have diagnostic value. For that, LAMP2 expression was further validated by ELISA in RBCs lysates. As shown in Figure 4*C*, LAMP2 expression level on M1 samples was significantly higher than CFC or M0 samples (*p* = 0.04 and 0.02 respectively, Mann–Whitney test), whereas no differences were observed between CFC and the M0 cohort. Additionally, M1 samples showed higher LAMP2 expression levels when compared with all the other samples together (CFC or M0 samples) (*p* = 0.01, Wilcoxon test) (Fig. 4*D*). Next, to evaluate LAMP2 levels as a diagnostic test for metastatic BC, receiver-operating characteristic analysis was performed including CFC (n = 20), nonmetastatic (M0, n = 24), and metastatic patients (M1, n = 15). The area under the curve value was 0.71, with 76% sensitivity and 62% specificity, thus LAMP2 expression levels of the RBCs were able to discriminate metastatic BC patients (Fig. 4*E*). Besides, to obtain a more robust predictive model, those blood test parameters that were altered in metastatic BC patients, as RDW and hematocrit, were included. This logistic regression model increased the discriminatory potential of metastatic BC patients (sensitivity = 92.3%, specificity = 80.5%, area under the curve = 0.89) (Fig. 4*F*), with a total success rate of 83.33% (Fig. 4*G*).

Since LAMP2 is ubiquitously expressed in white blood cells, plasma samples were also tested by ELISA assay. No differences were found between BC patients and CFC in LAMP2 protein levels in plasma, proving the specific RBCs origin of the increased LAMP2 expression (Fig. 4*H*).

## Discussion

RBCs are traditionally considered exclusively gas transporters. However, the constant renewal of circulating RBCs consumes large amounts of energy daily, suggesting a central role for RBCs in human physiology and homeostasis (26). RBC’s average life span is 120 days, which may reflect the systemic imbalance in the body. Consequently, they act as markers of specific diseases and their evolution (27, 28). Altered RBCs have been described in several diseases such as diabetes and Alzheimer’s, multiple sclerosis (29), and rheumatoid arthritis (30). Furthermore, the crosstalk between RBCs and immune cells has been described to cause the progression of atherosclerotic disease (31, 32, 33). More recently, RBCs have been described to bind cell-free DNA, which leads to phagocytosis of RBCs and innate immune activation in pathological settings (34), uncovering a previously unappreciated role of RBCs as critical players in inflammation. Thus, RBCs have been proposed as a circulating organ impacting systemic metabolic homeostasis and other cell functions. This can foster the development of novel therapeutic interventions in pathological hypoxemia, inflammation, neurodegenerative diseases, aging, and cancer (35). In cancer patients, small-scale studies have been published, pointing to protein alterations in RBCs (36, 37, 38) or RBC interaction with tumor cells (39). However, no reported studies are published using massive proteomics analysis. Hence, the major finding of this work is that RBCs from BC have a differential proteome profile compared with CFC, and it also varies among patients with different tumor stages.

Interestingly, in patients with BC, a higher presence of embryonic/fetal Hbs such as HBE1, theta (HBAT) or HBZ were observed. These Hbs correspond to <1% of the total in adult mammals. Different types of Hbs (including HBE1 and HBZ) have been detected in glioblastoma cell lines (40), while HBE1 has been associated with radio-resistance in colorectal cancer cells (41). Furthermore, tumor cells can directly generate erythroid cells, composed predominantly of embryonic Hbs, to obtain oxygen in response to hypoxia (42, 43). Indeed, embryonic or fetal Hbs have a higher oxygen affinity (44), which could give them an advantage in oxygen transfer in conditions of hypoxia or anemia, conditions frequently seen in cancer patients (45, 46). In this regard, HBD has been involved in the regulation of fetus–adult Hb switch (47) and was detected in hematopoietic stem cells and hepatocarcinoma cells (48, 49). In CTCs, hemoglobin beta has been related to cell survival (50). In our study, HBD was identified by both proteomic approaches, with an increase in protein levels of RBCs both in nonmetastatic and metastatic BC patients. Indeed, higher levels of the different HB chains (including hemoglobin beta) have been observed in BC patients by proteomics. However, this is contrary to the blood test data, in which Hb is lower in patients, especially in metastatic BC patients. One explanation could be that the blood test determines total HB levels and does not provide information on whether there is an imbalance of the HB chains that make up HBA, HBA2, or HBF. In addition, metastatic BC patients showed lower hematocrit compared with CFC or nonmetastatic BC, confirming previously reported works (51, 52). We also found high values of RDW in the metastatic BC patients, in accordance with a reported meta-analysis and other studies that have shown that RDW may be a potential prognostic marker in cancer patients (8, 9, 10, 11, 53, 54). Likewise, it has been suggested that an increment on immature RBCs in the circulation could be the underlying reason behind the rise in the RDW value in cancer patients. However, there is some controversy, since some authors support the relationship between RDW and cancer is a reflection of the effect that inflammation and oxidative stress cause on RBCs and that are also cancer risk factors. The alteration of blood parameters can have a multifactorial origin, including treatment. M0 samples were obtained before surgery or neoadjuvant therapy while M1 samples were collected before therapy initiation. Thus, in this work, the potential influence of treatment is negligible.

Our findings indicate that the proteins differentially expressed on the BC samples were mainly related to proteasome, exocytosis, and amino acid metabolism. Therefore, we have identified two clinically relevant proteins directly related to these pathways such as LAMP2 and PNP. The LAMP2 was specifically overexpressed in RBCs, and higher levels were associated with shorter outcomes in metastatic BC patients. Accordingly, with our data, LAMP2 expression levels could act as a reliable biomarker for diagnosing metastasis in BC, and its specificity and sensibility are increased when it is combined with the blood test parameters which account for RBCs status, hematocrit, and RDW. Interestingly, the capability of these parameters to diagnose metastasis in this patient cohort themselves is lower than LAMP2 alone (data not shown). Regarding PNP, which is highly expressed in physiological RBCs, shows higher levels on RBCs from BC patients. Interestingly, the PNP level is even higher in patients with visceral metastasis. This could be related to differences in metabolic demands depending on the site of metastasis, as has previously reported (55).

The amino acid metabolism, which is altered in many types of cancer has been linked as a hallmark of malignancy (56). Amino acids increase the metabolic rate of tumor cells and promote survival and proliferation (57, 58). The existence of an amino acid exchange between RBCs and different tissues or their ability to quickly absorb and release amino acids has been proved (59, 60, 61, 62), supporting the role of RBCs as amino acid transporters between organs. Thus, the presence of high concentrations of amino acids in RBCs without being required by internal metabolic processes could be explained if they serve as a metabolic supply to tumor cells. This is in accordance with the presence of other proteins like endopeptidases, proteasome, and chaperones that are involved in protein synthesis and degradation. Indeed, proteasome activity was identified in mature RBCs (63). Dekel *et al.* (64) demonstrated that *Plasmodium falciparum* parasites, cultured in fresh blood from a human donor, secrete extracellular vesicles that contain functional 20S proteasome complexes and that these can reshape the cytoskeleton of naïve RBCs. Under normal physiological conditions, RBC-derived extracellular vesicles (EVs) constitute 7.3% of EVs in whole blood, indicating that RBCs are one of the main sources of EVs in peripheral blood (65). Several studies have shown that EVs play key roles in cell-to-cell communication, and in cancer, they can regulate metastasis tropism. In addition, a study in children with neuroblastoma, described altered erythropoiesis, suggested that primary tumor cells produce effects at distance and that the observed impairment could be mediated by extracellular vesicles released by the tumor cells (66). In addition, EVs are the main miRNA carriers in the circulatory system. miRNAs play regulatory roles in the terminal differentiation process of RBCs, and they accumulate in mature RBCs (65).

LAMP2 is an important regulator in the effective maturation of both autophagosomes and phagosomes (67). Besides, it is involved in macroautophagy, chaperone-mediated autophagy, and receptor trafficking (68, 69) and has been involved in cell survival in BC (70). It has also been proposed that it may play a role in the activation of quiescent hematopoietic stem cells through chaperone-mediated autophagy (71). Thus, the detection of LAMP2 on RBCs from metastatic BC patients may be related to impaired hematopoiesis, a phenomena frequently found on cancer patients (72). During the reticulocyte maturation process, LAMP2 is lost, and reticulocytes are thought to use the exosome release pathway for the removal of proteins that are misplaced in the mature erythrocytes. It has been suggested that autophagy and exocytosis cooperate in the process of organelle elimination, forming hybrid vesicles by the fusion of the outer membrane of the autophagosome and the endosome derived from the plasma membrane (73). In this regard and due to the presence of LAMP2 or other endosome markers as Rab5 (see PXD030936), we can speculate that RBCs from metastatic BC patients could maintain a modified endosomal–lysosomal system which would support protein turnover as a reservoir of amino acids for tumor cells. Furthermore, in the RBCs population with altered features, the identified proteins point to an increase mainly in extracellular cellular components, reinforcing our hypothesis. In spite of emerging evidence which indicates that tumors alter the host hematopoietic system and induce the biased differentiation of myeloid cells, how this modification of normal hematopoiesis happens is poorly understood (74). Indeed, the blood test abnormalities found in BC patients mirror the stress in erythropoiesis on the bone marrow.

One limitation of this study is that the GO analysis should be read cautiously since it includes the set of proteins newly identified in RBCs that are yet to be included in the databases as RBCs related. Human RBCs proteome has been previously described by different groups using diverse technologies (24, 25, 75). However, the LC-MS/MS in DDA mode performed in the present study, combined with a SWATH-MS quantitative, can give an additional proteomic profile of RBCs and also give quantitative values of the proteins. Importantly, 43 identified proteins in CFC RBCs were not in public databases, pointing to the need of extended studies in this regard to be up-to-date in hand with the new technology developments. This study does not take into account the patients comorbidities which could be affecting the proteomic profile of the RBCs.

During the metastatic cascade, there are microenvironmental and systemic processes that contribute to cancer regulation, such as immune surveillance (76, 77). RBCs are now considered cells with systemic influence and a dynamic relationship with their environment (26). RBCs circulate in plasma together with a diversity of cells; therefore, many alterations within and on RBCs may result from contact with plasma proteins or soluble factors (including drugs), with substances released from activated cells and likewise with nucleated cells as CTCs and other circulating tumor material. Although RBCs were historically considered inert to regulatory signals from other cells, they are well equipped with the machinery required for intercellular communication. Thus, as recently published, they are capable to regulate the biological processes of neighboring cells, becoming a novel regulatory cell (28). The observed changes in RBCs from BC patients compared with CFC are probably multifactorial where the tumor microenvironment may be having a part, together with inflammation (78). Hence, more research is needed in this direction. Remarkably, RBCs are easy to access, highly abundant, and a systemically distributed biological component (26); thus, RBC proteins can be useful biomarkers for cancer monitoring of BC patients and potentially of other tumor types and may constitute a new kid on the block in the liquid biopsy field.

## Conclusions

In conclusion, this study revealed that the presence of a tumor modifies the RBC proteome and points to the value of RBC proteins in the prognosis and diagnosis of metastatic BC in a noninvasive way. Our data provide new information that could open a new study path in the context of disseminated disease.

## Data Availability

The mass spectrometry proteomics data have been deposited to the ProteomeXchange Consortium *via* the PRIDE partner repository with the dataset identifier PXD030936. A related patent entitled “*In vitro* method for the diagnosis or prognosis of breast cancer” (PCT/EP21382448.5) has been deposited.

## Supplemental data

This article contains supplemental data.

## Conflict of interest

R. L.-L. reports grants and personal fees from Roche, Merck, AstraZeneca, Bayer, Pharmamar, and Leo and personal fees and nonfinancial support from Bristol-Myers Squibb and Novartis, outside of the submitted work. The other authors declare no conflict of interest.

## Supplemental Data

Supplemental Table S1 List of patients and CFC showing concentration of RBCs protein (μg/μl), hematocrit value, and subtype information. **Supplemental Table S2**. Summary of numbers of peptides identified from RBCs extracts from pools of cancer-free controls (CFC), M0, and M1 BC patients by shotgun. **Supplemental Tables S3–S5**. List of peptides of CFC, M0, or M1 pooled samples from shotgun analysis. **Supplemental Table S6**. Library of proteins identified by SWATH-MS in CFC samples. **Supplemental Table S7**. List of differentially expressed proteins by SWATH-MS. **Supplemental Table S8**. List of potential novel proteins identified by Swath-Ms or shotgun compared with reported RBCs and platelets databases.
