# Supplementary material for: Red Blood Cells Protein Profile Is Modified in Breast Cancer Patients
Source: Mol Cell Proteomics. 2022 Oct 28;21(12):100435. doi: 10.1016/j.mcpro.2022.100435 (PMC9713370; doi:10.1016/j.mcpro.2022.100435)
Supplement: Supplemental Methods and Materials [file mmc2.docx]

***Protein digestion***

- 1. To make global protein identification and quantification, an equal amount of protein (over 100 µg), from RBCs of 84 samples (53 Breast Cancer (BC) patients and 33 donors (cancer-free controls)) was loaded on a 10% SDS-PAGE gel. The run was stopped as soon as the front had penetrated 3 mm into the resolving gel(1,2). The protein band was detected by Sypro-Ruby fluorescent staining (Lonza, Switzerland), excised, and processed for in-gel, manual tryptic digestion as described elsewhere(3), gel pieces were reduced with 10 mM dithiothreitol (Sigma-Aldrich, St. Louis, MO) in 50 mM ammonium bicarbonate (Sigma-Aldrich, St. Louis, MO) and alkylated with 55 mM iodoacetamide (Sigma- Aldrich, St. Louis, MO) in 50 mM ammonium bicarbonate. Then, the gel pieces were rinsed with 50 mM ammonium bicarbonate in 50% methanol (HPLC grade, Scharlau, Barcelona, Spain), dehydrated by addition of acetonitrile (HPLC grade, Scharlau, Barcelona, Spain), and dried in a SpeedVac. Modified porcine trypsin (Promega, Madison, WI, USA) was added to the dry gel pieces at a final concentration of 20 ng/μl in 20mM ammonium bicarbonate, incubating them at 37 °C for 16 h. Peptides were extracted thrice by 20 min incubation in 40 μL of 60% acetonitrile in 0.5% HCOOH. The resulting peptide extracts were pooled, concentrated in a SpeedVac, and stored at −20 °C.

***Mass spectrometric analysis (DDA acquisition)***

Digested peptides (over 4 µg of each sample) were separated using Reverse Phase Chromatography. The gradient was created using a micro liquid chromatography system (Eksigent Technologies nanoLC 400, Sciex) coupled to a high-speed Triple TOF 6600 mass spectrometer (Sciex) with a microflow source. The analytical column was a silica-based reversed-phase column Eksigent C18 150 × 0.30 mm, 3 mm particle size and 120 Å pore size (Eksigent, Sciex). The trap column was an YMC-TRIART C18 (YMC Technologies, Teknokroma) with a 3 mm particle size and 120 Å pore size, switched online with the analytical column. The loading pump delivered a solution of 0.1% formic acid in water at 10 µl/min. The micro-pump generated a flow rate of 5 µl/min and was operated under gradient elution conditions. For that, 0.1% formic acid in water was used as mobile phase A, and 0.1% formic acid in acetonitrile as mobile phase B. Peptides were separated using a 90 minutes gradient ranging from 2% to 90% mobile phase B.

Data acquisition was performed in a TripleTOF 6600 System (Sciex, Foster City, CA) using a Data dependent workflow (DDA). Source and interface conditions were the following: ionspray voltage floating (ISVF) 5500 V, curtain gas (CUR) 25, collision energy (CE) 10 and ion source gas 1 (GS1) 25. The instrument was operated with Analyst TF 1.7.1 software (Sciex, USA). Switching criteria were set to ions greater than mass to charge ratio (m/z) 350 and smaller than m/z 1400 with a charge state of 2–5, mass tolerance 250 ppm and an abundance threshold of more than 200 counts (cps). Former target ions were excluded for 15 s. The instrument was automatically calibrated every 4 hours using external calibrant tryptic peptides from PepCalMix.

***Data Analysis***

After MS/MS analysis, data files processing was done using ProteinPilot^TM^5.0.1 software from Sciex which uses the algorithm Paragon^TM^ for database search and Progroup^TM^ for data grouping. Data were searched using a Human-specific Uniprot database (UniProt release 2020_01 Published on February 26, 2020. 20365 Human proteins), specifying iodoacetamide as Cys alkylation as variable modification and metionin oxidation as fixed modification. The false discovery rate was calculated using a non-linear fitting method displaying only those results that reported a1% Global false discovery rate or better(4).

***Protein quantification by SWATH (Sequential Window Acquisition of all Theoretical Mass Spectra)***

**Creation of the spectral library**

To build the MS/MS spectral libraries, the peptide solutions were analyzed by a shotgun **data-dependent acquisition (DDA)** approach using **micro-LC-MS/MS**. To obtain a good representation of the peptides and proteins present in all samples, pooled vials of samples from each group were prepared using equal mixtures of the original samples. One 4 μL of each pool (Breast Cancer (BC) patients: (M0 and M1), and Cancer-free controls (CFC)) were separated into a micro-LC system Ekspert nLC425 (Eksigen, Dublin, CA, USA) using an Eksigent C18 150 × 0.30 mm, 3 mm particle size and 120 Å pore size (Eksigent, Sciex) at a flow rate of 5µL/min. Water and ACN, both containing 0.1% formic acid, were used as solvents A and B, respectively. The gradient run consisted of 5% to 95% B for 30 min, 5 min at 90% B and finally 5 min at 5% B for column equilibration, for a total run time of 40 min. As the peptides eluted, they were directly injected into a hybrid quadrupole-TOF mass spectrometer Triple TOF 6600 (Sciex, Redwood City, CA, USA) operated with a data-dependent acquisition system in positive ion mode. A Micro source (Sciex) was used for the interface between microLC and MS, with an application of 2600 V voltage. The acquisition mode consisted of a 250 ms survey (MS scan) MS1 from 400 to 1250 m/z followed by an (MS/MS analysis) MS2 analysis scan from 100 to 1500 m/z (25 ms acquisition time) of the top 65 precursor ions from the survey scan, for a total cycle time of 2.8 s. The fragmented precursors were then added to a dynamic exclusion list for 15 s; any singly charged ions were excluded from the (MS/MS analysis) MS2 analysis.

The peptide and protein identifications were performed using Protein Pilot software (version 5.0.1, Sciex) with a Data was searched using a Human-specific Uniprot database (UniProt release 2020_01 Published on February 26, 2020. 20365 Human proteins), specifying iodoacetamide as Cys alkylation as variable modification and metionin oxidation as fixed modification. The false discovery rate (FDR) was set to 1 for both peptides and proteins. The MS2 spectra (MS/MS spectra) of the identified peptides were then used to generate the spectral library for SWATH peak extraction using the add-in for PeakView Software (version 2.2, Sciex) MS/MS^ALL^ with SWATH Acquisition MicroApp (version 2.0, Sciex). Peptides with a confidence score above 99% (as obtained from the Protein Pilot database search) were included in the spectral library.

**Relative quantification by SWATH acquisition**

SWATH (Sequential Window Acquisition of all Theoretical Mass Spectra) – MS acquisition was performed on a TripleTOF® 6600 LC-MS/MS system (Sciex). FourμL of Peptides from each individual RBCs samples were analyzed using a data-independent acquisition (IDA) method making 3 technical replicate for each sample. Each sample (4 μL) was analyzed using the LC-MS equipment and LC gradient described above for building the spectral library but instead using the SWATH-MS acquisition method. The method consisted of repeating a cycle that consisted of the acquisition of 65 TOF (MS/MS scans) MS2 (400 to 1500 m/z, high sensitivity mode, 50 ms acquisition time) of overlapping sequential precursor isolation windows of variable width (1 m/z overlap) covering the 400 to 1250 m/z mass range with a previous TOF MS1 scan (400 to 1500 m/z, 50 ms acquisition time) for each cycle. Total cycle time was 6.3 s. For each sample set, the width of the 65 variable windows was optimized according to the ion density found in the DDA runs using a SWATH variable window calculator worksheet from Sciex.

**Data analysis**

The targeted data extraction of the fragment ion chromatogram traces from the SWATH runs was performed by PeakView (version 2.2, Sciex) using the SWATH Acquisition MicroApp (version 2.0). This application processed the data using the spectral library created from the shotgun data. Up to 10 peptides per protein and 7 fragments per peptide were selected, based on signal intensity; any shared and modified peptides was excluded from the processing. Five minute windows and 30 ppm widths were used to extract the ion chromatograms; SWATH quantization was attempted for all proteins in the ion library that were identified by ProteinPilot with an FDR below 1%. The retention times from the peptides that were selected for each protein were realigned in each run according to the iRT peptides present in the samples and eluted along the whole time axis. The extracted ion chromatograms were then generated for each selected fragment ion; the peak areas for the protein were obtained by summing the peak areas from 10 peptides (MS1 scan) and 7 corresponding fragment ions (MS2 scan) from each peptide. PeakView computed an FDR and a score for each assigned peptide according to the chromatographic and spectra components; only peptides with an FDR below 1 % were used for protein quantization. Protein quantization was calculated by adding the peak areas of the corresponding peptides.

The integrated peak areas (processed. mrkvw files from PeakView) were directly exported to the MarkerView software (Sciex) for relative quantitative analysis. The export generated three files containing quantitative information about individual ions, the summed intensity of different ions for a particular peptide and the summed intensity of different peptides for a particular protein. MarkerView uses processing algorithms that accurately find chromatographic and spectral peaks direct from the raw SWATH data. Data alignment by MarkerView compensates for minor variations in both mass and retention time values, ensuring that identical compounds in different samples are accurately compared to one another. To control for possible uneven sample loss across the different samples during the sample preparation process, we performed a global normalization based on the total sum of all the peak areas extracted from all the peptides and transitions across the replicates of each sample(5). Unsupervised multivariate statistical analysis using principal component analysis (PCA) was performed to compare the data across the samples, the average MS peak area of each protein was derived from the replicates of the SWATH-MS of each sample followed by Student’s *t*-test analysis using the MarkerView software for comparison among the samples based on the averaged area sums of all the transitions derived for each protein. The *t*-test will indicate how well each variable distinguishes the two groups, reported as a *p*-value. For the protein/peptide library, its set of differentially expressed proteins (*p*-value *<*0.05) up-regulated or down-regulated proteins were selected.

SWATH-MS has been demonstrated to be a translational and a valuable tool in different fields being successfully applied by our group in a large variety of studies (6,7-19).

**References**

1. Bonzon-Kulichenko E, Pérez-Hernández D, Núñez E, Martínez-Acedo P, Navarro P, Trevisan-Herraz M, et al. A robust method for quantitative high-throughput analysis of proteomes by 18O labeling. Mol Cell Proteomics. 2011;10(1).

2. Perez-Hernandez D, Gutiérrez-Vázquez C, Jorge I, López-Martín S, Ursa A, Sánchez-Madrid F, et al. The intracellular interactome of tetraspanin-enriched microdomains reveals their function as sorting machineries toward exosomes. J Biol Chem. 2013;288(17).

3. Shevchenko A, Wilm M, Vorm O, Mann M. Mass Spectrometric Sequencing of Proteins from Silver-Stained Polyacrylamide Gels. Anal Chem. 1996 Jan;68(5):850–8.

4. Shilov I V., Seymourt SL, Patel AA, Loboda A, Tang WH, Keating SP, et al. The paragon algorithm, a next generation search engine that uses sequence temperature values sequence temperature values and feature probabilities to identify peptides from tandem mass spectra. Mol Cell Proteomics. 2007;6(9).

5. Karpievitch Y V., Dabney AR, Smith RD. Normalization and missing value imputation for label-free LC-MS analysis. BMC Bioinformatics. 2012;13 Suppl 16.

6. Novelle MG, Bravo SB, Deshons M, Iglesias C, García-Vence M, Annells R, et al. Impact of liver-specific GLUT8 silencing on fructose-induced inflammation and omega oxidation. iScience. 2021;24(2).

7. Tamara C, Nerea L-B, Belén BS, Alberto M-V, Aurelio S, Iván C, et al. HUMAN OBESE WHITE ADIPOSE TISSUE SHEDS DEPOT-SPECIFIC EXTRACELLULAR VESICLES AND REVEALS CANDIDATE BIOMARKERS FOR MONITORING OBESITY AND ITS COMORBIDITIES. Transl Res. 2021 Jan;

8. M G-V, MDP C-V, A S-F, R A, A B de la I, A O-G, et al. Protein Extraction From FFPE Kidney Tissue Samples: A Review of the Literature and Characterization of Techniques. Front Med. 2021 May;8.

9. Chantada-Vázquez M del P, García Vence M, Serna A, Núñez C, Bravo SB. SWATH-MS Protocols in Human Diseases. In: Methods in Molecular Biology. 2021.

10. JV Á, SB B, MP C-V, S B-G, C C, O L-S, et al. Plasma Proteomic Analysis in Morquio A Disease. Int J Mol Sci. 2021 Jun;22(11).

11. L O-O, E A-L, M P-M, F L-G, MC GF, L D, et al. Circulating Extracellular Vesicle Proteins and MicroRNA Profiles in Subcortical and Cortical-Subcortical Ischaemic Stroke. Biomedicines. 2021 Jul;9(7).

12. Peñas-Martínez J, Barrachina MN, Cuenca-Zamora EJ, Luengo-Gil G, Bravo SB, Caparrós-Pérez E, et al. Qualitative and quantitative comparison of plasma exosomes from neonates and adults. Int J Mol Sci. 2021;22(4).

13. Camino T, Lago-Baameiro N, Bravo SB, Sueiro A, Couto I, Santos F, et al. Vesicles Shed by Pathological Murine Adipocytes Spread Pathology: Characterization and Functional Role of Insulin Resistant/Hypertrophied Adiposomes. Int J Mol Sci. 2020;21(6).

14. Álvarez VJ, Bravo SB, Chantada-Vazquez MP, Colón C, De Castro MJ, Morales M, et al. Characterization of new proteomic biomarker candidates in mucopolysaccharidosis type IVA. Int J Mol Sci. 2021;22(1).

15. da Silva Lima N, Fondevila MF, Nóvoa E, Buqué X, Mercado-Gómez M, Gallet S, et al. Inhibition of ATG3 ameliorates liver steatosis by increasing mitochondrial function. J Hepatol. 2021 Sep;

16. Gonzalez-Rellan MJ, Fondevila MF, Fernandez U, Rodríguez A, Varela-Rey M, Veyrat-Durebex C, et al. O-GlcNAcylated p53 in the liver modulates hepatic glucose production. Nat Commun. 2021;12(1).

17. López-López M, Regueiro U, Bravo SB, Chantada-Vázquez M del P, Varela-Fernández R, Ávila-Gómez P, et al. Tear proteomics in keratoconus: A quantitative SWATH-MS analysis. Investig Ophthalmol Vis Sci. 2021;62(10).

18. Anfray C, Mainini F, Digifico E, Maeda A, Sironi M, Erreni M, et al. Intratumoral combination therapy with poly(I:C) and resiquimod synergistically triggers tumor-associated macrophages for effective systemic antitumoral immunity. J Immunother cancer. 2021 Sep;9(9).

19. Gómez-Cid L, López-Donaire ML, Velasco D, Marín V, González MI, Salinas B, et al. Cardiac extracellular matrix hydrogel enriched with polyethylene glycol presents improved gelation time and increased on-target site retention of extracellular vesicles. Int J Mol Sci. 2021;22(17).
